# Design of Efficient Exciplex Emitters by Decreasing the Energy Gap Between the Local Excited Triplet (^3^LE) State of the Acceptor and the Charge Transfer (CT) States of the Exciplex

**DOI:** 10.3389/fchem.2019.00188

**Published:** 2019-04-09

**Authors:** Xiaofang Wei, Yanwei Liu, Taiping Hu, Zhiyi Li, Jianjun Liu, Ruifang Wang, Honglei Gao, Xiaoxiao Hu, Guanhao Liu, Pengfei Wang, Chun-sing Lee, Ying Wang

**Affiliations:** ^1^Key Laboratory of Photochemical Conversion and Optoelectronic Materials, Technical Institute of Physics and Chemistry, Chinese Academy of Sciences, Beijing, China; ^2^School of Future Technology, University of Chinese Academy of Sciences, Beijing, China; ^3^Beijing National Laboratory for Molecular Sciences, Key Laboratory of Organic Solids, Institute of Chemistry, Chinese Academy of Sciences, Beijing, China; ^4^Center of Super-Diamond and Advanced Films (COSDAF), City University of Hong Kong, Hong Kong, Hong Kong

**Keywords:** organic light emitting diode (OLED), pure thermally activated delayed fluorescence (TADF), exciplex, thixanthone (TX) derivatives, energy gap

## Abstract

A series of thermally activated delayed fluorescence (TADF) exciplex based on the TX-TerPy were constructed. The electronic coupling between the triplet local excited states (^3^LE) of the donors and acceptor and the charge transfer states had a great influence on the triplet exciton harvesting and Φ_PL_. Herein, based on this strategy, three donor molecules TAPC, TCTA, and m-MTDATA were selected. The local triplet excited state (^3^LE) of the three donors are 2.93, 2.72 and 2.52 eV in pure films. And the ^3^LE of TX-TerPy is 2.69 eV in polystyrene film. The energy gap between the singlet charge transfer (^1^CT) states of TAPC:TX-TerPy (7:1), TCTA:TX-TerPy (7:1) and the ^3^LE of TX-TerPy are 0.30 eV and 0.20 eV. Finally, the Φ_PL_ of TAPC:TX-TerPy (7:1) and TCTA:TX-TerPy (7:1) are 65.2 and 69.6%. When we changed the doping concentration of the exciplex from 15% to 50%, the ratio of the triplet decreased, and Φ_PL_ decreased by half, perhaps due to the increased energy gap between ^1^CT and ^3^LE. Therefore, optimizing the ^1^CT, ^3^CT, and ^3^LE facilitated the efficient exciplex TADF molecules.

**Graphical Abstract:**
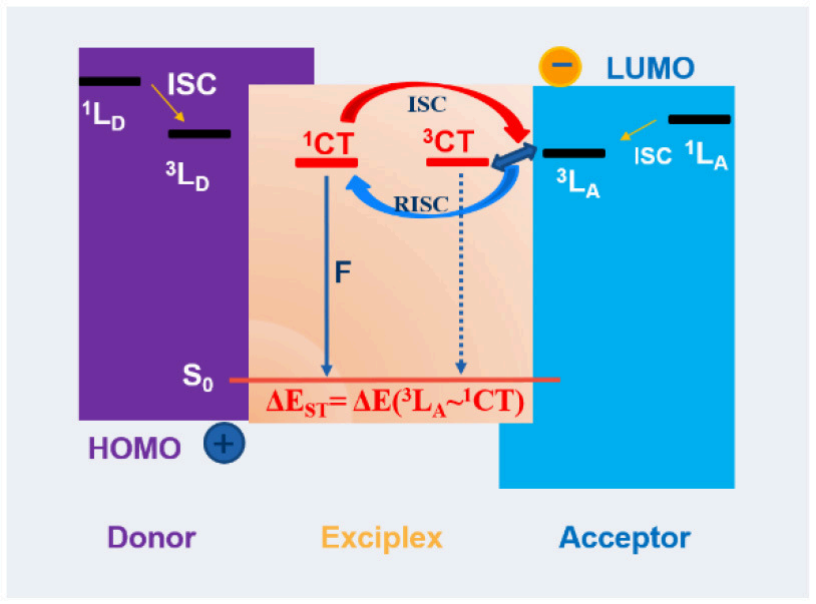
Design and prediction of the highly efficient exciplex emitters is desirable to optimize the ^1^CT, ^3^CT of the exciplex and ^3^LE of the donor or accepter. Here, we studied the newly molecule TX-TerPy as the acceptor blended with TAPC, TCTA and m-MTDATA. The energy gap between the ^1^CT and ^3^LE in TCTA: 15 wt% TX-TerPy blends film is as small as 0.19 eV. So the Φ_PL_ is the highest one and nearly 100% triplet exciton can be obtained.

## Introduction

Organic light-emitting devices (OLED) have been widely studied due to their promising applications in large displays and solid-state lighting (Sasabe and Kido, 2013). During recent years, thermally activated delayed fluorescence (TADF) in OLEDs have been desirable due to low-cost fabrication and 100% exciton utilization through the effective intersystem crossing (ISC) and the reverse intersystem crossing (RISC) from triplet state (T_1_) to singlet state (S_1_), to harness the 25% singlet exciton and the 75% triplet excitons (Tao et al., 2014; Lee et al., 2015; Cui et al., 2016; Wong and Zysman-Colman, 2017; Yang et al., 2017). Hence, an internal quantum efficiency (IQE) of 100% for the TADF-based OLEDs can be achieved. The progress based on the TADF emitters reported that the EQEs exceed 30% (Lee et al., 2015; Lin et al., 2016), which is comparable for the phosphorescence OLEDs (PHOLEDs) and much higher than the device based on the traditional fluorescence.

Besides pure TADF emitters, exciplex is an alternative kind of efficient TADF OLED emitters. The efficient separation of the highest occupied molecular orbital (HOMO) and the lowest unoccupied molecular orbital (LUMO) in exciplex located on two different molecules resulted in a smaller Δ*E*_ST_ (ca. 0–50 meV) which allows the non-radiative different kind of exciplex system showing efficient TADF property have been reporetd. The efficient separation of the highest occupied molecular orbital (HOMO) and lowest unoccupied molecular orbital (LUMO) in exciplex located on two different molecules results of the smaller Δ*E*_ST_ (ca. 0–50 meV), which allows the non-radiative T_1_ to radiative S_1_ via RISC. Many different kinds of exciplex systems showing efficient TADF property have been reported (Graves et al., 2014; Deotare et al., 2015; Liu et al., 2015a,b,c; Wu et al., 2017; Duan et al., 2018; Lin et al., 2018). The EQEs based on them have exceeded 5%, which is the limit for a device based on fluorescent emitters. Moreover, many groups reported that the PHOLEDs and TADF based OLEDs used exciplexes as hosts demonstrating low operating voltages, low efficiency roll-off, and high efficiencies (Li et al., 2014; Liu et al., 2016; Kim et al., 2017; Moon et al., 2017; Shih et al., 2018). However, the exciplex systems generally cannot avoid the relatively lower photoluminescence (PL) quantum yield (PLQY) than the theoretical limit, which is perhaps due to the non-radiative transition of excited state (Goushi et al., 2012; Hung et al., 2013; Park et al., 2013). Exciplex emitters are needed to further efficiency enhancement. Recently, many reports have described that the spin orbital coupling (SOC) is formally forbidden between singlet and triplet CT (^1^CT and ^3^CT) states. Despite the weakness of the hyperfine coupling (HFC), the ^1^CT state can only form a strong couple to a close lying local triplet state (^3^LE) (dos Santos et al., 2016; Etherington et al., 2016; Santos et al., 2016; Samanta et al., 2017; Mamada et al., 2018) despite of the hyperfine coupling (HFC) between ^1^CT and ^3^CT, the ^1^CT state will strongly couple to a close lying local triplet state (^3^LE) since the SOC between ^1^CT and ^3^CT are assumed to be forbidden (dos Santos et al., 2016; Etherington et al., 2016; Santos et al., 2016; Samanta et al., 2017; Mamada et al., 2018). The gap between the ^1^CT and ^3^LE is the real ΔE_ST_ which requires thermal energy for triplets to cross over to a singlet state meaning that a closed ^3^LE is indispensable for the ISC and RISC as shown in Graphical Abstract. However, a few reports have clarified the specific value of the energy gap between ^1^CT and ^3^LE state. Meanwhile, the triplet exciton energy levels should be higher than those of the CT states, in order to prevent “back transfer” loss to a lower-energy triplet exciton of the donors or acceptors (Deotare et al., 2015; Liu et al., 2015c; Mamada et al., 2018).

**Figure 1:**
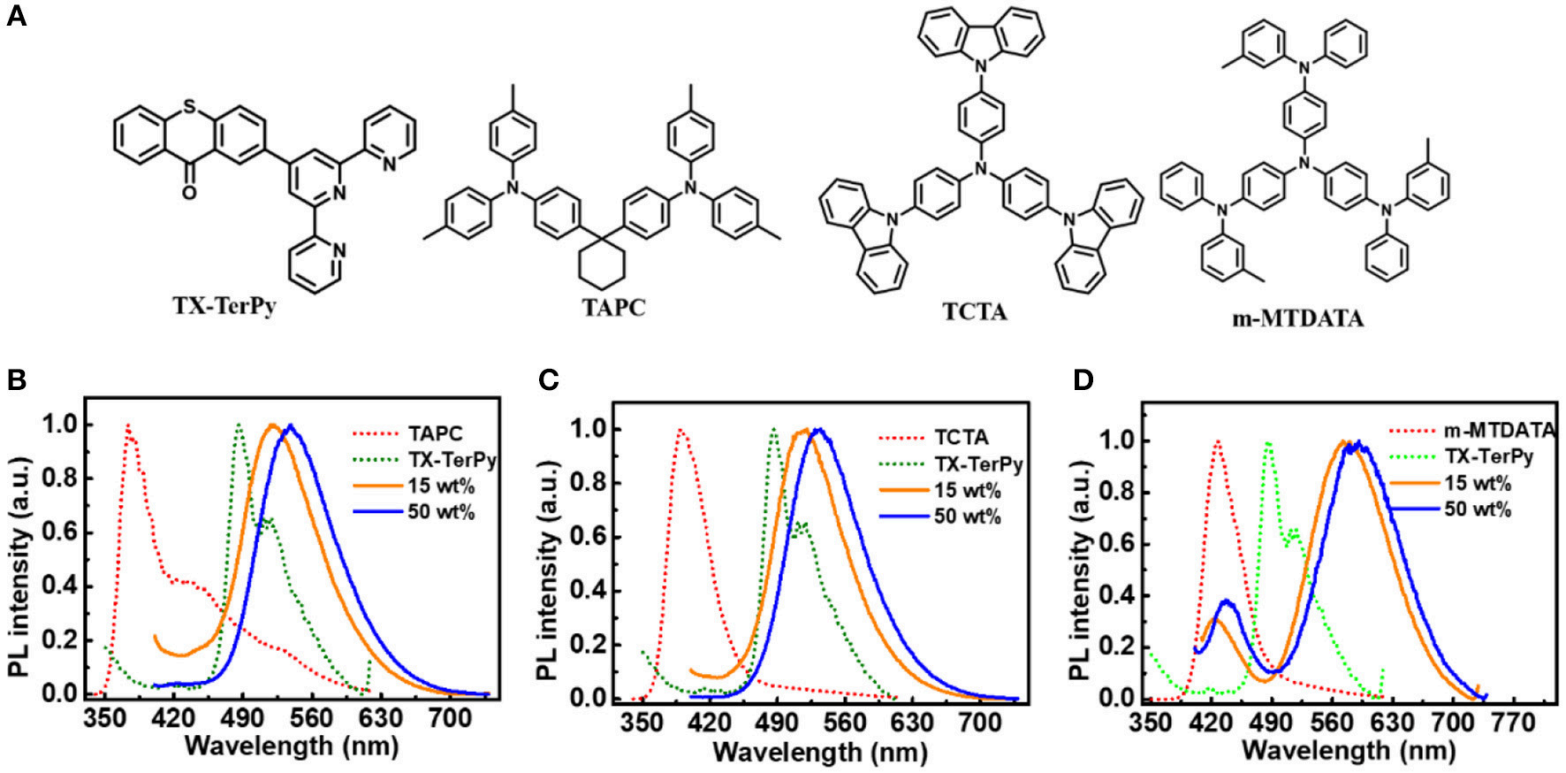
Molecular structures and photoluminescence (PL) spectra of deposited films. **(A)** Molecular structures of TX-TerPy, TAPC, TCTA, and m-MTDATA; **(B)** PL spectra of TAPC, TX-TerPy, TAPC:TX-TerPy (7:1) and TAPC:TX-TerPy (1:1); **(C)** TCTA, TX-TerPy, TCTA:TX-TerPy (7:1) and TCTA:TX-TerPy (1:1); **(D)** m-MTDATA, TX-TerPy, m-MTDATA:TX-TerPy (7:1) and m-MTDATA:TX-TerPy (1:1).

Herein, three exciplex emitters are designed by adopting our newly molecules Thixanthone (TX) derivatives 2-([2,2′:6′,2″-terpyridin]-4′-yl)-9H-thioxanthen-9-one(TX-TerPy) as the acceptor, with the common donors containing the triphenylamine unit to detect the factors leading to high PLQY. The donors are TAPC, TCTA and m-MTDATA, which are common hole-transporting material, namely di-[4-(N,N-di-p-tolyl-amino)-phenyl]cyclohexane, 4,4′,4“-Tris(carbazol-9-yl)triphenylamine and 4,4′,4″-Tris(N-3-methylphenyl-N-phenyl-amino) triphenylamine, respectively (Graves et al., 2014; Liu et al., 2015c; dos Santos et al., 2016; Shih et al., 2018). The detail chemical structures of these four compounds are shown in Figure 1A. The three blended films show green and yellow exciplex emissions which are red shifted greatly compared to their corresponding individual molecules. The TAPC:TX-TerPy, TCTA:TX-TerPy and m-MTDATA:TX-TerPy exciplex system exhibit photoluminescence quantum efficiency (Φ_PL_) of 65.2, 69.6%, and 10.7% in N_2_ atmosphere, respectively, when the doping concentration of TX-TerPy is 15%. In TAPC:TX-TerPy, TCTA:TX-TerPy, the effective exciton confinement of the constituting molecules avoid the exciton leakage due to the high T_1_ state. Despite the high T_1_ energy level of the two individual molecules of TX-TerPy and m-MTDATA, the energy leakage from the exciplex to the T_1_ excited state of m-MTDATA has occurred, which results in a low Φ_PL_ for m-MTDATA:TX-TerPy. Significantly, the ^3^LE of TX-TerPy is more close to the ^1^CT of TCTA:TX-TerPy compared to TAPC:TX-TerPy, which are 0.19 eV and 0.28 eV, respectively. Thus, the more effective exciton harvesting can be guaranteed by the small energy gap between ^3^LE of TX-TerPy and the ^1^CT of the exciplex system, especially for TCTA:TX-TerPy. Based on the high Φ_PL_ of TAPC:TX-TerPy and TCTA:TX-TerPy, OLEDs are fabricated to investigate their electrochemical characteristics. The green emitting devices based on TAPC:TX-TerPy and TCTA:TX-TerPy present turn-on voltages of 3.5 V and 3.4 V as well as high maximum current (CE) and power efficiencies (PE) and external quantum efficiencies (EQE) of 7.08 and 8.29 %, 22.18 cd A^−1^ and 25.83 cd A^−1^, 21.07 lm W^−1^ and 23.19 lm W^−1^, respectively. And the luminance have reached to 6000 m^−2^ and 8000 cd m^−2^, respectively. The result is higher than those of the traditional fluorescence emitters but slower than the expected result from the Φ_PL_. Therefore, the efficiency of devices can be further improved by optimizing the carrier balance and carrier traps. Thus, in premise of efficient energy transfer from the host to exciplex emitters, the doping concentration of the acceptor should be as low as possible to shift up the ^1^CT of the exciplex blends. The result above demonstrated a simple way to design the exciplex blends to obtain nearly 100% triplet exciton by decreasing the energy gap between ^3^LE states of the acceptor and the ^1^CT of the exciplex emitters.

## Results and Discussion

### Synthesis, Thermal Properties, and Photophysical Properties

TX unit is promising as an acceptor unit to construct the efficient pure TADF (Wang et al., 2014; Li et al., 2016) While, TX derivatives have never been used as the acceptor molecule to construct the exciplex, we functionalized the TX unit by bridging it along with the terpyridy by Suzuki coupling reactions, an electron-withdrawing group, to obtain the molecules TX-TerPy. In exciplex, molecules containing nitrogen heterocycles are always preferred acceptors due to their deep HOMO levels (Shan et al., 2016; Deksnys et al., 2017; Nagai et al., 2017; Wu et al., 2018). TX-TerPy was successfully synthesized as shown in Scheme S1 and fully characterized by ^1^H NMR spectroscopy (Figure S1), High-resolution EI-mass spectrometry (Figure S2), and satisfactory element analysis. Thermal gravimetric analysis (TGA) and differential scanning calorimetry (DCS) were also carried out at a heat rate of 10°C min^−1^ under a nitrogen atmosphere to observe the thermally stability, respectively. It has an excellent thermal properties with decomposition temperature of 338.8°C, corresponding to 5 wt% weight loss, which fulfill the requirement of film preparation via vacuum deposition (Figure S3) and the glass transition temperature (Tg) of the bulk amorphous film was 141.3°C. Moreover, Figure S4 showed the UV/Vis absorption bands and photoluminancence (PL) spectrum of it in different solvents from the low polarity toluene to the high polarity THF. The bands at around 380 nm is the weak intramolecular charge transfer (ICT) transition derived from the TX unit to terpyridy unit. And the faint red shift of the PL emission in different solvent shows the weak ICT property in the excited state. Then the fluorescent and phosphorescent emissions were examined at 77 K in oxygen-free 2-MeTHF solution and in pure film (shown in Figures S5, S9). And the details are shown in Tables S1, S2. They exhibited the large Δ*E*_ST_ of 0.38 and 0.48 eV, respectively. The fluorescence and phosphorescence at 77 k both showed fine structure, which is both attributed to the ^1^LE and ^3^LE in solution or in pure film. And the decay lifetime of TX-TerPy is only 5.6 ns. Thus, TX-TerPy is a normal fluorescent molecule. ^3^LE of the molecule might depend on the polarity of the surrounding media, we not only detected it in neat film in more polar conditions, but also doped the TX-TerPy into polystyrene (PS) to detect it in less polar conditions. Thus, we estimated the S_1_ and T_1_ energy from the maxima emission of the fluorescent and phosphorescent in the blended films of PS at 77 K, as shown in Figure S6. The S_1_ and T_1_ are 3.03 eV and 2.69 eV, respectively. The details are all shown in Table 1. The energy offset between the donor's HOMO and acceptor's LUMO drive the electron transfer that leads to exciplex formation (Liu et al., 2011; Jankus et al., 2014; Lee et al., 2017; Song et al., 2017). We chose the donor molecules with suitable HOMO or LUMO offset with TX-TerPy and higher ^3^LE energy level in order to avoid the exciton leakage which was discussed previously (Liu et al., 2011). Meanwhile, choosing the donors with different T_1_ energy is an important way to manipulate the exciplex energy level in order to obtain the efficient TADF exciplex emitters. Next three hole-transporting materials of TAPC, TCTA and m-MTDATA were selected to act as the donors.

**Table 1:** Summary of Photophysical properties of exciplex films.

**Acceptor**	**Donor**	**Conc. ^a^**	**Φ_PL_^b^**	**Φ_PL_^c^**	**Φ_prompt_^d^**	**Φ_delayed_^e^**	**λ_em_^f^**	**E_S1_^g^**	**E_T1_^g^**	**Δ*E*(^1^CT−^3^CT)^h^**
		**[wt%]**	**[%]**	**[%]**	**[%]**	**[%]**	**[nm]**	**[eV]**	**[eV]**	**[eV]**
	TAPC	15	59.2	65.2	1.90	63.30	520	2.79	2.75	0.02
		50	37.8	38.5	0.57	37.93	539	2.67	2.65	0.02
TX-TerPy	TCTA	15	59.0	69.6	0.02	69.58	521	2.80	2.77	0.03
		50	46.6	47.6	1.43	46.17	535	2.69	2.66	0.03
	m-MTDATA	15	9.6	10.7	1.22	9.48	572	2.15	2.62	/
		50	5.3	6.2	0.84	5.36	590	2.15	2.45	/

a Acceptor concentration.

b Absolute PL quantum yield (Φ_PL_) measured under air flow in an integrating sphere at room temperature.

c Absolute PL quantum yield (Φ_PL_) measured under N_**2**_ flow in an integrating sphere at room temperature.

d The ratio of the prompt component of Φ_PL._

e The ratio of the delayed component of Φ_PL._

f Measured at room temperature.

g Singlet (E_S_) and triplet (E_T_) excited energies estimated from the maximum wavelengths of fluorescence and phosphorescence spectra at 77 K in doped film, respectively

h *Δ*E*(^1^CT−^3^CT)=ΔES - ΔET*.

### Exciplex Formation

The three donor molecules are mixed with TX-TerPy by weight of 1:1 to form the exciplexes. Figures 1B–D shows the PL spectra of the neat films of TX-TerPy, TAPC, TCTA and m-MTDATA and their corresponding exciplex emitters. The emission for TAPC:TX-TerPy, TCTA:TX-TerPy and m-MTDATA:TX-TerPy exciplex system show the maximum peaks at 538.6 nm, 534.6 nm and 590.6 nm, whereas those for TX-TerPy, TAPC, TCTA and m-MTDATA display the wavelength of 486.6, 373.6, 389.6 and 428.6 nm, respectively. The PL spectrum of the blended films are redshift by 52, 48 and 104 nm relative to the acceptor molecule. Especially for m-MTDATA:TX-TerPy, the interaction between m-MTDATA and TX-TerPy are stronger than the other two exciplex emitters, perhaps since that m-MTDATA holds three triphenylanime units. And the full width at half maximum (FWHM) of the three exciplex emissions is much broader than those of the constituting molecules. The large redshift emission band with broad, featureless structure compared to the emission of the corresponding neat films confirms the formation of exciplex in each of the blend films. The absorption bands of the doped films exhibit only the linear combination of the individual absorption of the donors and acceptors molecules suggesting that there is not a new-state transition in them (as shown in Figure S7). The results above all indicate the formation of the exciplex between the individual molecules.

(1) $$\begin{array}{r} E_{exciplex}=E_{D}^{ox}-E_{A}^{red}+U_{dest}-U_{stab}-\Delta H_{e}^{sol}+0.32\text{eV} \end{array}$$

*E*_*exciplex*_ is the exciplex photon energy. In exciplex, the intermolecular charge transfer derived from the donor and the acceptor leads that the *E*_*exciplex*_ has a linear relationship with the oxidation potential of the donor ($E_{D}^{ox}$) and the reduction potential of the acceptor ($E_{A}^{red}$), as shown in equation (1) (Cocchi et al., 2006; Liu et al., 2015c; Sarma and Wong, 2018). For an exciplex, U_dest_ and U_stab_ is nearly 0, which is, the stabilization and destabilization effects of the exciplex formation (Jankus et al., 2014). $\Delta H_{e}^{sol}$is the enthalpy of solvation which is nearly 0.17 ± 0.07 eV. Exciplex formation requires a charge transfer from the donor's HOMO to acceptor's LUMO, thus the considerable off-set of nearly 0.5 eV between the LUMO levels of the donor and acceptor should be satisfied (Jankus et al., 2014). The *E*_*exciplex*_ of TAPC:TX-TerPy, TCTA:TX-TerPy, and m-MTDATA:TX-TerPy are estimated to be 2.30, 2.32 and 2.10 eV. The HOMO energies of the four molecules are determined by Ultraviolet photoelectron spectroscopy (UPS) measurement (Figure S8), they were −6.02, −5.52, −5.95, and −5.14 eV, respectively. The LUMO levels were then calculated with the optical band gap determined from their absorption spectra. They are −3.15, −1.98, −2.57, and −1.96 eV. Then, $E_{D}^{ox}-E_{A}^{red}$ is estimated to be 2.37, 2.80, and 1.99 eV, close to the *E*_*exciplex*_ of the three exciplex system, which are indicated the emission of the blend film is formed between the individual donors and the acceptor. For the TAPC:TX-TerPy, *E*_*exciplex*_ is 0.07 eV lower than their related $E_{D}^{ox}-E_{A}^{red}$.Whereas, TCTA:TX-TerPy is 0.48 eV larger than their corresponding $E_{D}^{ox}-E_{A}^{red}$, which indicates the exciplex has a degree charge transfer of < 0.5. The state contains some heteroexcimer and some full ion pair state. For m-MTDATA:TX-TerPy, hv_max_ is 0.09 eV larger than their related $E_{D}^{ox}-E_{A}^{red}$. The TCTA:TX-TerPy should have good PLQY due to the strongly mixed LE and CT character.

**Figure 2:**
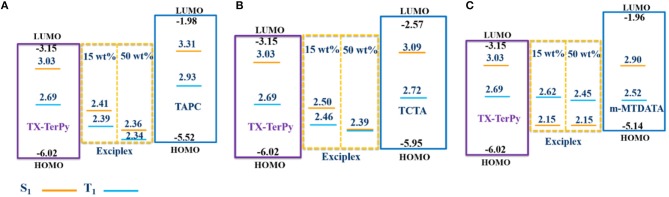
HOMO, LUMO, and excited-state energies of TX-TerPy, TAPC, TCTA, m-MTDATA and the exciplex systems. **(A)** HOMO, LUMO, and S_1_ and T_1_ excited-state energies of TX-TerPy, TAPC and exciplex systems at different doping concentration; **(B)** HOMO, LUMO, and S_1_ and T_1_ excited-state energies of TX-TerPy, TCTA and exciplex systems at different doping concentration; **(C)** HOMO, LUMO, and S1and T1excited-state energies of TX-TerPy, m-MTDATA and exciplex systems at different doping concentration.

In order to quantify the Δ *E*_ST_ experimentally, as shown in Figure S5 and Table S1, we investigated the fluorescence and phosphorescence spectra of the individual donors in 2-MeTHF at room temperature and 77K. And we also investigated the fluorescence and phosphorescence spectra of TAPC:TX-TerPy, TCTA:TX-TerPy and m-MTDATA:TX-TerPy in doped films (7:1 and 1:1) (Figure S11) and fluorescence and phosphorescence spectra of the individual pure films (Figure S9) at 77 k. The details are shown in Table S2. The details of the S_1_ and T_1_ excited state are shown in Figure 2 and Table 2. The TAPC:TX-TerPy and TCTA:TX-TerPy blended films both show the unstructured fluorescence and phosphorescence emission at 77 k. From the maximum emission peaks of the fluorescence and phosphorescence spectra, the S_1_ and T_1_ state of TAPC:TX-TerPy were calculated to be 2.36 and 2.34 eV, respectively. So the S_1_-T_1_ energy gap is 0.02 eV. The blended film displays fluorescence at 77 k with features from both the host and the exciplex emission, but also the unstructured phosphorescence spectra. It is perhaps due to the inefficient energy transfer from TAPC or TX-TerPy to exciplex at low temperature. As for TCTA:TX-TerPy, the S_1_ and T_1_ are 2.39 and 2.39 eV, which are both lower than that of TAPC:TX-TerPy. And the energy gap is nearly 0. Meanwhile, the phosphorescence of the donors show characteristic vibrational structures with the first highest peaks at 423.6 nm for TAPC, 456.6 nm for TCTA, 491.6 nm for m-MTDATA, so the related T_1_ energy is 2.93, 2.72 eV and 2.52 eV, respectively. The ^3^LE of the donors are all higher than the T_1_ state of the corresponding exciplex emitters, so the energy transferring from the exciplex to the donors can be avoided. Surprisingly, the phosphorescence of m-MTDATA:TX-TerPy is arising from m-MTDATA, despite that the T_1_ energy of m-MTDATA (2.52 eV) are higher than S_1_ energy (2.15 eV) of m-MTDATA:TX-TerPy, perhaps due to the inefficient reverse energy transfer from m-MTDATA to exciplex. Since the ^1^CT, ^3^CT, and ^3^LE should be match with each other, we optimize the exciplexes at different doping concentrations, which will have an influence on ^1^CT and ^3^CT of the exciplex emitters. As shown in Figure S11, the S_1_ and T_1_ energy of TAPC:TX-TerPy emitters shift up above 0.05 eV when we decrease the concentration of TX-TerPy from 50 to 15 wt%. That will benefit to decrease the energy gap between ^1^CT of the exciplex and ^3^LE of the acceptor and donor. As for TCTA:TX-TerPy (7:1), the S_1_ and T_1_ shift up above 0.11eV, larger than TAPC:TX-TerPy. But for the individual molecules, the ^3^LE changed a little, perhaps due to the insensitive environment dependence of it. That indicated that the RISC process of TCTA:TX-TerPy is more efficient than TAPC:TX-TerPy.

**Table 2:** Summary of OLEDs Performance.

**Exciplex emitters ^a^**	**L_max_ [cd m^−2^]^b^**	**V_on_ [V]^c^**	**CE_max_ [cd A^−1^]^d^**	**PE_max_ [lm W^−1^]^d^**	**EQE_max_ [%]^d^**	**CIE (x,y)^e^**
TAPC:TX-TerPy	6,000	3.4	22.18	21.07	7.08	(0.39, 0.54)
TCTA:TX-TerPy	8,000	3.5	25.83	23.19	8.29	(0.40, 0.54)

a The devices based on TAPC:20%TX-TerPy in a structure of ITO/ TAPC (35 nm)/mCP (10 nm) /Exciplex (EML) (15 nm)/TmPyPB (50 nm)/LiF (0.9 nm)/Al (100 nm). TCTA:TX-TerPy (1:1) in a structure of ITO / α-NPD (20 nm)/ TCTA (10 nm)/ mCP (10 nm) /Exciplex (EML) (15 nm) / TmPyPB (50 nm) /LiF (0.9 nm)/Al (100 nm).

b The maximum luminance.

c Turn-on voltage at 1 cd m^−2.^

d The maximum efficiencies of CE (cd A^−1^), PE (lm W^−1^) and EQE (%).

e *The Commission Internationale de L'Eclairage coordinates recorded at 7 V*.

### Photoluminescence Decays

**Figure 3:**
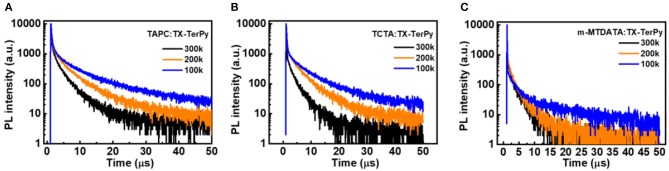
Temperature dependence of photoluminescence (PL) characteristics of the exciplex films. **(A)** PL decay curves of a TAPC:50 wt% TX-TerPy film at 300 K, 200 K, and 100 K; **(B)** PL decay curves of a TCTA:50 wt% TX-TerPy film at 300 K, 200 K, and 100 K; **(C)** PL decay curves of a m-MTDATA:50 wt% TX-TerPy film at 300 K, 200 K, and 100 K.

In order to look inside into the TADF property of the three exciplex emitters, we conducted a transient photoluminescence experiment. As the different doping concentrations will tune the energy levels of the ^1^CT energy of the blend films, we further studied the transient lifetime of the different constituting concentration of TX-TerPy. The choice of the different donors have a great influence on the Φ_PL_ (Table 1). When the doping concentration had the weight of 1:1, the Φ_PL_ are 38.5%, 47.6, and 6.2%, respectively. Whilst, the doping concentration for TAPC:TX-TerPy, TCTA:TX-TerPy and m-MTDATA:TX-TerPy had the weight of 7:1 Φ_PL_ of 65.2, 69.6, and 10.7% were for the exciplex emitters. Clearly, TAPC:TX-TerPy and TCTA:TX-TerPy both show higher Φ_PL_ at the weight of 7:1 and are nearly two times higher than their corresponding doping concentrations of 50%. Notably, the TCTA: 15 wt% TX-TerPy demonstrates that the highest values among the six exciplex system is smaller than those of the other exciplexes as a result of the energy gap between ^3^LE of TX-TerPy and ^1^CT of TCTA:TX-TerPy As shown in Figure S10, the films of the pristine TX-TerPy, TAPC, TCTA, and m-MTDATA all show PL decays with the lifetime of around 2 ns at room temperature. In Figure S13, the transient PL decay spectral of the three exciplex emitters (1:1) at room temperature in N_2_ flow all demonstrated a clear two-order or three-order exponential decays. The prompt ones refers to the relaxation from S_1_ to S_0_ and the delayed one refers to the delayed fluorescence (DF) and other persistent luminescence in the exciplex emitters. The DF refers the triplet excitons up-converse to the S_1_ state via the RISC process, then decay to S_0_. We divided them into two components in order to simplify the comparison. Surprisingly, they all showed a strong delayed emission, as shown in Figure 3. Then according to the PL intensity of the different components of TAPC:TX-TerPy (1:1), the Φ_PL_ at room temperature can be divided into 1.5% for the prompt component and 98.5% for the delay component, which indicated that the most triplet exciton can be obtained. For the TCTA:TX-TerPy, the delayed component can be as high as 97.0%, which is due to the smaller energy between ^3^LE of TX-TerPy and the S_1_ and T_1_ energy level of the exciplex. Although the Φ_PL_ of m-MTDATA:TX-Terpy is very low, the delayed component is still as large as 86.5%. The prompt fluorescence decay of the TAPC:TX-TerPy, TCTA:TX-TerPy and m-MTDATA:TX-TerPy blended films is 150, 220 and 170 ns, which is much longer than the individual films. As for the constituting concentration of 15% of the three exciplex blended films, the delayed components are higher than that of 50%, which is 97.1% for TAPC:TX-TerPy, 99.9% for TCTA:TX-TerPy and 88.6% for m-MTDATA:TX-TerPy. These are higher than the exciplex systems mentioned previously. The detail of the prompt fluorescence efficiency (Φ_prompt_) and the delayed emission efficiency (Φ_delayed_) of the six exciplex emitters are shown in Table 1.

Then, the temperature dependence transient decay and PL emission of the three of the exciplex emitters (weight of 1:1) were performed from 300 K to 100 K to determine the nature of the delayed component (Figure 3). As shown in Figure 3, the PL intensity of the three exciplexes all increased with the decreasing temperature from 300 to 100 K (Figure S12), as do the PL intensity of the delay component, perhaps due to the emergence of the phosphorescence or the effect of singlet-triplet recycling. And the two component decay lifetimes of the longer component also varied as the temperature decreased. The lifetime of the delayed fluorescence of the three exciplexes nearly remained the same, namely the second component of the decay, but their ratio decreased a lot as the temperature decreased. On the contrary, for TAPC:TX-TerPy, the longer decay compound lifetime increased from 4.06 to 9.03 us. While TAPC:TX-TerPy increased form3.57 to 8.93 us, and for m-MTDATA:TX-TerPy, 2.78 to 15.02 us as the temperature varied from 300 K to 100 K. The ratio was at least 79% at 300 K which was beneficial for the triplet exciton to obtain. This does not only verify the origins of the delayed ones, deriving from the recursive S_1_ to S_0_ transition via the RISC of the T_1_ to S_1_ by absorbing exoteric heat energy, but also verifies the emergence of the other long decay lifetimes, which was due to the small active energies.

### Electroluminescence Properties

**Figure 4:**
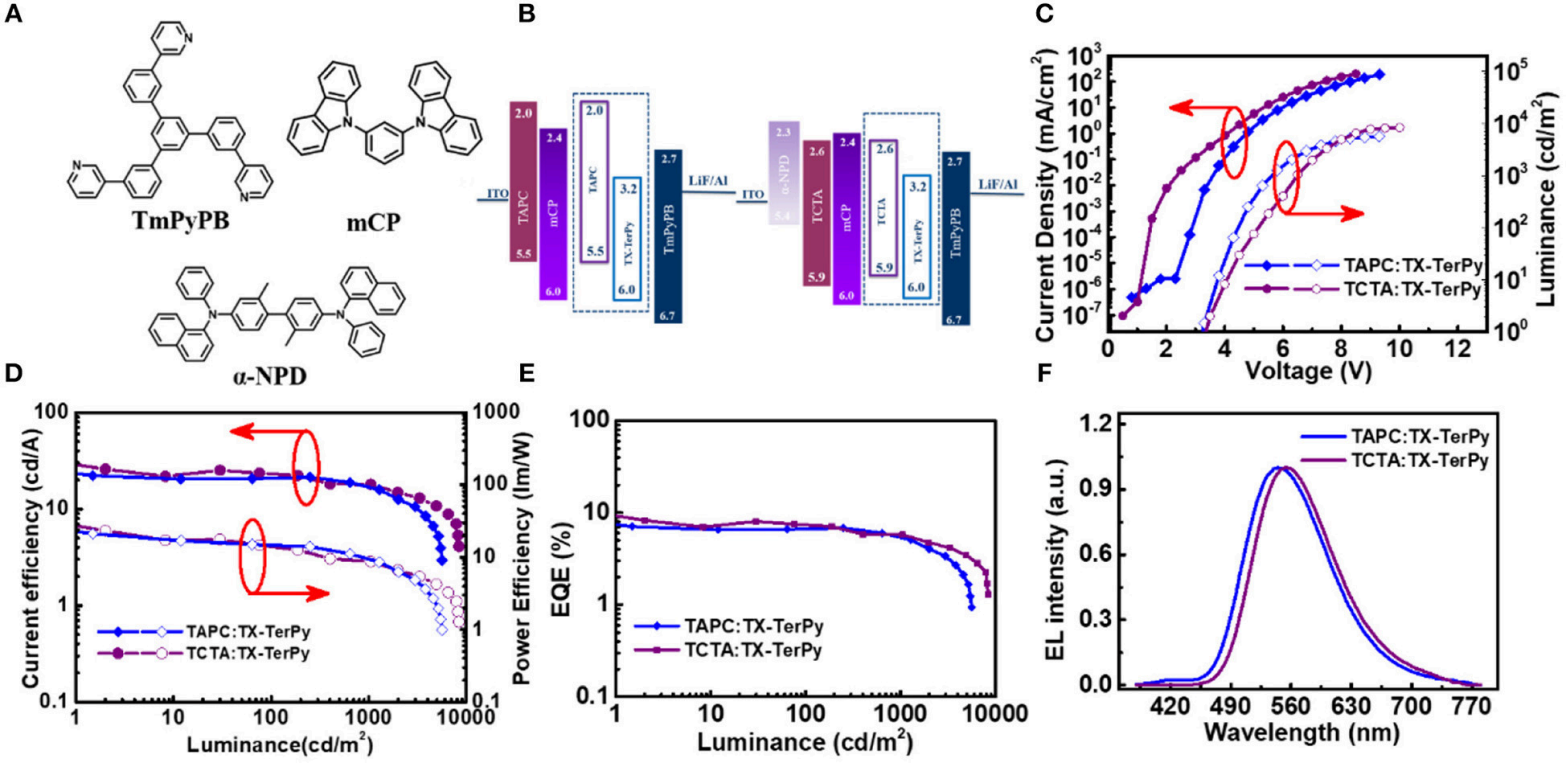
Device characteristics of the devices based on TAPC:TX-TerPy and TCTA:TX-TerPy. **(A)** Molecular structure, and **(B)** the energy level of the devices; **(C)** Current density-voltage-luminance characteristics; **(D)** Current efficiency and power efficiency-luminance characteristics; **(E)** EQE-luminance characteristics; and **(F)** EL spectra.

In order to further investigate the electrochemical property of the exciplex blend film based on TAPC:TX-TerPy and TCTA:TX-TerPy, OLED devices were fabricated with the following structures: indium tin oxide (ITO)/TAPC(35 nm)/1,3-Bis(carbazol-9-yl)benzene (mCP) (10 nm)/TAPC:TX-TerPy (15 nm)/1,3,5-tri(m-pyrid-3-yl-phenyl)benzene (TmPyPB) (50 nm)/LiF (1.0 nm)/Al (100 nm) and ITO/ N,N′-Bis(naphthalen-1-yl)-N,N′-bis(phenyl)-2,2′-dimethylbenzidine (α-NPD) (20 nm)/TCTA (10 nm)/mCP (10 nm)/TCTA:TX-TerPy (15 nm)/TmPyPB (55 nm)/LiF (0.9 nm)/Al (100 nm). Figures 4A,B show the molecule structure and schematic diagram of the OLEDs and energy levels of the organic materials. Among them, α-NPD acts as the hole injection layer, TAPC or TCTA as the hole transport layer, mCP as the electron-blocking layer, and TmPyPB as the electron transporting and hole blocking layer. The devices based on TAPC:TX-TerPy and TCTA:TX-TerPy show green emission centered at 544 and 556 nm, which are red shifted corresponding to their PL spectrum. In order to optimize the device performance, the donor to acceptor weight ratio (D/A, w/w) in the active layer and the thickness of the active layer were modulated. As depicted in Figures 4C–F, the performance of the device with the optical D/A ratio (5:1) for TAPC:TX-TerPy and 1:1 for TCTA:TX-TerPy showed the highest efficiency. The OLED device based on TAPC:TX-TerPy and TCTA:TX-TerPy show the current efficiency (CE) of 22.13 cd A^−1^ and 25.83 cd A^−1^, the power efficiency of 21.07 lm W^−1^ and 23.19 lm W^−1^ and a high external quantum efficiency (EQE) of 7.08 and 8.29%, respectively. The details are shown in Table 2. The devices show the EQE is much lower than those expected from their Φ_PL_ (D/A ratio 1:1). The efficiency of the devices can be further improved by the optimization of the carrier balance, carrier traps and doping concentration. The device performance will be studied later. The 7.08 and 8.29% of the EQE based on this exciplex is much higher than the theoretical limit of 5% of the conventional fluorescence emitters. This will prove that the efficient triplet is harvesting in exciplex.

## Conclusions

In summary, the three hole transporting molecules containing triphenylamine unit with different T_1_ energy were selected as the donors to form exciplex with the newly molecule TX-TerPy. They all displayed a second order decay or third decay transient PL decay curves with the prompt and delayed component or long decay compound. Finally, the exciplex based on TCTA displayed the highest Φ_PL_ due to the appropriate HOMO of TCTA and LUMO of TX-TerPy, the appropriate ^1^CT, ^3^CT, and ^3^LE energy level of them, especially the small energy gap between ^3^LE of TX-Tery and S_1_ of TCTA:TX-TerPy. The OLED device based on TAPC:TX-TerPy and TCTA:TX-TerPy displayed the CE of 22.13 and 25.83 cd A^−1^, the power efficiency of 21.07 and 23.19 lm W^−1^ and a high EQE of 7.08 and 8.29%, respectively. These findings highlight the optimized the ^1^CT, ^3^CT, and ^3^LE in facilitating the efficient exciplex TADF molecules. Further studies on the adjustment the energy of the three state will open a new way for high performance for OLED device efficiency.

## Author Contributions

XW, JL, and YW conceived the idea of the study, designed the experiment, and performed most of the optical measurement. XW, JL, RW, XH, and HG synthesized and characterized the molecules under the supervision of YW. YL, ZL, and GL fabricated and measured the devices. XW, YW, CL, and PW wrote the manuscript. TH answered the question about theoretical calculation in comments. All authors contributed to the scientific discussion.

### Conflict of Interest Statement

The authors declare that the research was conducted in the absence of any commercial or financial relationships that could be construed as a potential conflict of interest.
