# Supplementary material for: Design of Efficient Exciplex Emitters by Decreasing the Energy Gap Between the Local Excited Triplet (3LE) State of the Acceptor and the Charge Transfer (CT) States of the Exciplex
Source: Front Chem. 2019 Apr 9;7:188. doi: 10.3389/fchem.2019.00188 (PMC6465540; doi:10.3389/fchem.2019.00188)
Supplement: Supplementary file 1 [file Presentation_1.pdf]

Supplementary Material

Design of Efficient Exciplex Emitters by Decreasing the Energy Gap between the Local Excited Triplet（^{3}LE）State of the Acceptor and the Charge Transfer (CT) States of the Exciplex

Xiaofang Wei[1,3], Yanwei Liu[2,3], Taiping Hu,[2,3] Zhiyi Li[1,3], Jianjun Liu[1,3], Ruifang Wang[1,3], Honglei Gao[1,3], Xiaoxiao Hu[1,3], Guanhao Liu[1,3], Pengfei Wang[1,3], Chun-Sing Lee[4], Ying Wang[1,3]*

[1]Key Laboratory of Photochemical Conversion and Optoelectronic Materials, Technical Institute of Physics and Chemistry, Chinese Academy of Sciences, Beijing, 100190, China
[2]Beijing National Laboratory for Molecular Sciences, Key Laboratory of Organic Solids, Institute of Chemistry, Chinese Academy of Sciences, Beijing, 100190, China
[3]School of Future Technology, University of Chinese Academy of Sciences, Beijing, 100049, China
[4]Center of Super-Diamond and Advanced Films (COSDAF), City University of Hong Kong, Hong Kong

*** Correspondence:**
Corresponding Author
wangy@mail.ipc.ac.cn

Keywords: Organic light emitting diode (OLED), Pure Thermally activated delayed fluorescence (TADF), Exciplex, Thixanthone (TX) derivatives, Energy gap.

1. Materials and methods.

1.1 General methods. All solvents and materials were used as received from commercial suppliers without further purification. All reactions were monitored by thin layer chromatographic analysis on a pre-coated silica gel plate, which was visualized by a UV lamp at 254 or 365 nm. Flash column chromatography was performed on glass column of silica gel (100–200 mesh) and solvent ratios were expressed in volume to volume. ^{1}H for structural characterization were recorded on a NMR spectrometer (400 MHz for ^{1}H). All NMR measurements were conducted in $CDCl_3$ at room temperature. The NMR spectra were recorded on VaRIAN-GEMINI-300 and Bruker Avance Π-400 spectrometer at room temperature and tetramethylsilane (TMS) as an internal reference. Mass spectra were recorded using a BIFLEXIII MALDI-TOF mass spectrometer in EI mode. Chemical shifts were reported as parts per million in scale using the solvent residual peak as internal standard for ^{1}H and ^{13}C NMR. Coupling constants (*J*) were reported in Hertz (Hz). UV-vis spectra and fluorescence spectra were obtained with Hitachi U-3900 and F-4600 spectrophotometers, respectively. The phosphorescence spectra were measured in 2-MeTHF glass matrix at 77 K using a Hitachi F-4600 fluorescence spectrometer. The absolute fluorescence quantum yields of the solid films are measured with an integrating sphere. The DSC measurements were carried out using a TA Instruments DSC 2910 thermal analyzer at a heating rate of 10 °C min^{-1}. The TGA measurements were performed on a TA Instruments TGA 2050 thermal analyzer. The transient PL decay and temperature dependence of the films were measured using an Edinburgh Instruments FLS920 spectrometer. Ultraviolet Photoelectron Spectroscopy of the film were measured using an ESCALAB 250Xi analyzer.

1.2 Synthesis. All solvents and materials were used as received from commercial suppliers without further purification. Synthetic routes of these compounds are shown in scheme S1.

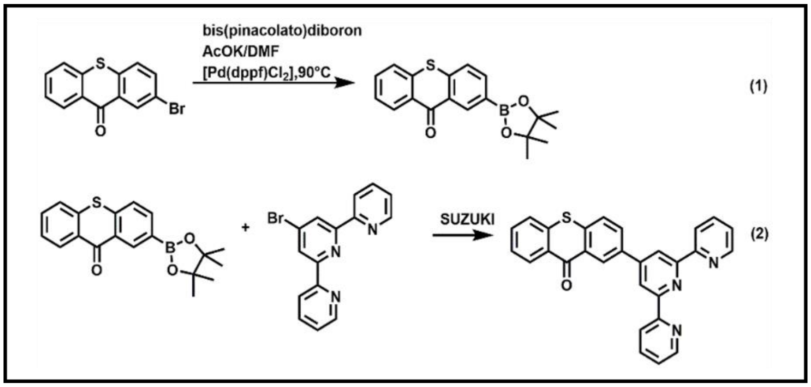

Scheme S1. Synthetic Routes of TX-TerPy.

Synthesis of 2-(4,4,5,5-tetramethyl-1,3,2-dioxaborolan-2-yl)-9H-thioxanthen-9-one (1):

2-bromo-9H-thioxanthen-9-one (1.45 g, 0.5 mmol), bis (pinacolato) diboron (5.06 g, 2 mmol) and potassium acetate (1.96 g, 2 mmol) were dissolved in 40 mL anhydrous DMF in a nitrogen atmosphere. Pd(pddf)Cl_2 (0.36 g, 0.48mmol) was then added to the reaction mixture. The reaction was carried at 90 for 6h and then cooled to the room temperature. Then it was poured into the water and filtrated. After dried, it was further purified by column chromatography on silica gel using a petroleum ether/dichloromethane mixture (3:1), and the pure product of compound was finally obtained. (1.18g, 0.35mmol, yield 70%). ^{1}H NMR (400 MHz, $CDCl_3$) δ 9.06 (s, 1H), 8.63 (d, *J* = 8.0 Hz, 1H), 7.98 (d, *J* = 7.9 Hz, 1H), 7.64 – 7.45 (m, 4H), 1.37 (s, 12H). calcd for $C_{19}H_{19}O_3BS$, 338.23; found: 338.23.

Synthesis of TX-TerPy:

4'-bromo-2,2':6',2''-terpyridine (2) (1.61 g, 0.5mmol), tetrakis(triphenylphosphine)palladium(0) (0.03 g, 0.027mol), 2 M K_2CO_3 (20 ml) and toluene (30ml) were added to a 100 ml three-necked flask under nitrogen. The 2-(4,4,5,5-tetramethyl-1,3,2-dioxaborolan-2-yl)-9H-thioxanthen-9-one (2.77g, 0.6mmol) was added to the solution and heated to 110 °C for 5 h. After cooling to room temperature, the reaction mixture was filtrated. The solvent was removed. The resulting crude product was passed through a flash column chromatograph (using CH_2Cl_2: PE, 1:5) to remove impurities. The yield of product was 60%.^{1}H NMR (400 MHz, $CDCl_3$) δ 9.18 (s, 1H), 8.89 (s, 2H), 8.82 – 8.59 (m, 5H), 8.22 (d, *J* = 8.4 Hz, 1H), 7.91 (t, *J* = 7.7 Hz, 2H), 7.75 (d, *J* = 8.4 Hz, 1H), 7.70 – 7.60 (m, 2H), 7.56 – 7.50 (m, 1H), 7.43 – 7.34 (m, 2H). Due to the limited solubility, the ^{13}C NMR is failed to obtain. EIMS m/z (%): calcd for $C_{28}H_{17}OSN_3$, 443.11; found: 443.11. Anal. calcd for $C_{37}H_{26}O_3Si$: C, 75.83; H, 3.86; N, 9.47; found: C,75.80; H, 3.83; N, 9.45.

1.3 Transient photophysical measurement. The transient photoluminance decay characteristics were measured using an Edinburgh Instruments FLS920 spectrometer. The temperature dependence experiment is conducted under low temperature refrigeration system from Advanced Research Systems Company. The transient decay curves were fitted with the multi-exponential equation:

$$I(t) = \sum_i A_i \exp(-t / \tau_i) \tag{1}$$

where A_i is the pre-exponential factor, τ_i is the decay time, and I(t) is the emission intensity. The Φ_{Prompt} and Φ_{TADF} were determined by using total PL quantum efficiency and the ratio between prompt and delayed components. The intensity ratio between prompt (r_1) and delayed (r_2, r_3) components were determined using emission life time (τ_1, τ_2, τ_3) and fitting parameter (A_1, A_2, A_3). Then, Φ_{Prompt} and $\Phi_{delayed}$ were determined using intensity ratio (r_1, r_2, r_3) and total emission quantum yield.

$$I_{prompt}/I_{delayed} = \Phi_{Prompt} / \Phi_{delayed} \quad (2)$$

1.4 Device fabrication. OLEDs were fabricated on patterned ITO-coated glass substrates with a sheet resistance of 15 Ω/□. Before device fabrication, the ITO glass substrates were sequentially cleaned with Decon 90, rinsed in de-ionized water, dried in an oven at 120°C, and treated with oxygen plasma. After that, the clean substrates were transferred into a vacuum deposition system with a base pressure better than 1×10^{-6} mbar for organic and metal deposition. The devices were fabricated by evaporating organic semiconductors onto the substrate sequentially with an evaporation rate of 1–2 Å s^{-1}. The cathode was completed through thermal deposition of LiF at a deposition rate of 0.1 Å s^{-1}, and then capped with Al metal through thermal evaporation at a rate of 10 Å s^{-1}. EL luminescence spectra and CIE color coordinates were measured with a Spectrascan PR655 photometer and the current-voltage characteristics were measured with a computer-controlled Keithley 2400 Source Meter with a calibrated optical power meter of Newport company (1936R) under ambient atmosphere.

1.5 Supplementary tables and figures

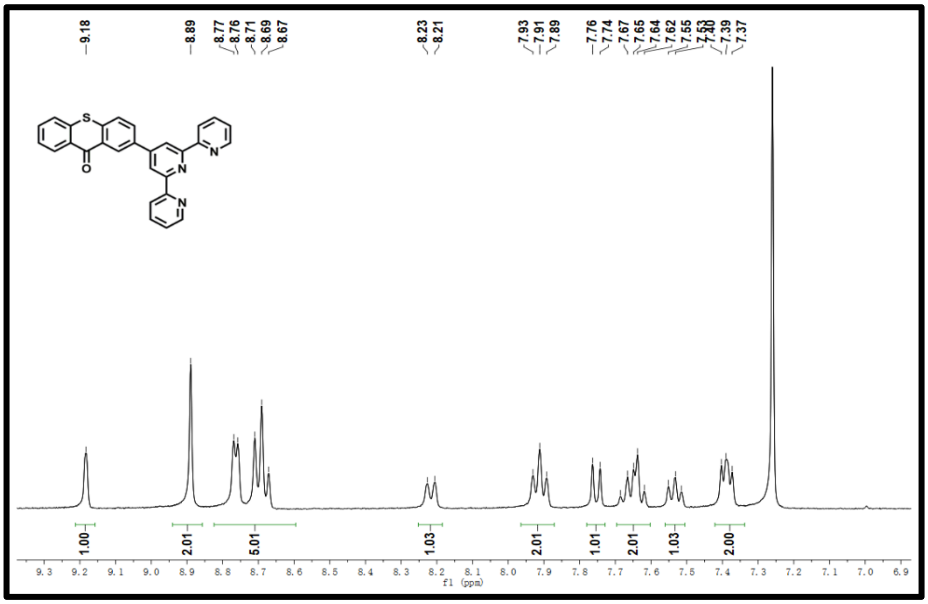

Figure S1. ^{1}H NMR spectrum of TX-TerPy.

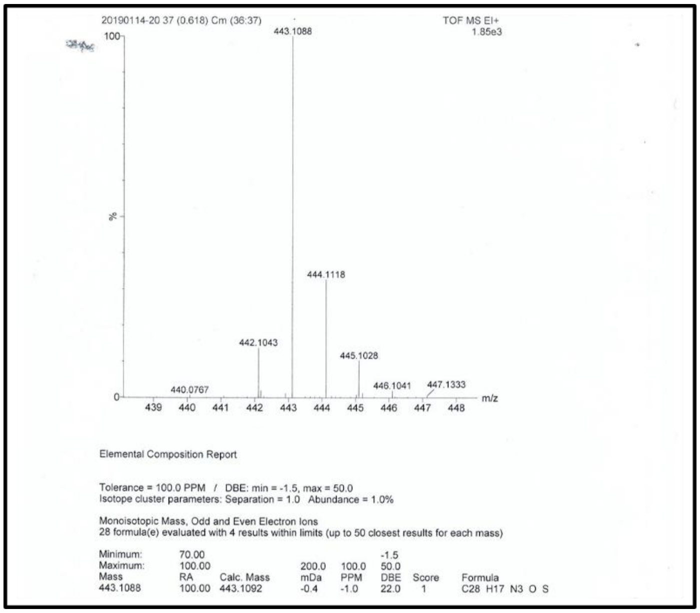

Figure S2. HRMS EI+ spectrum of TX-TerPy.

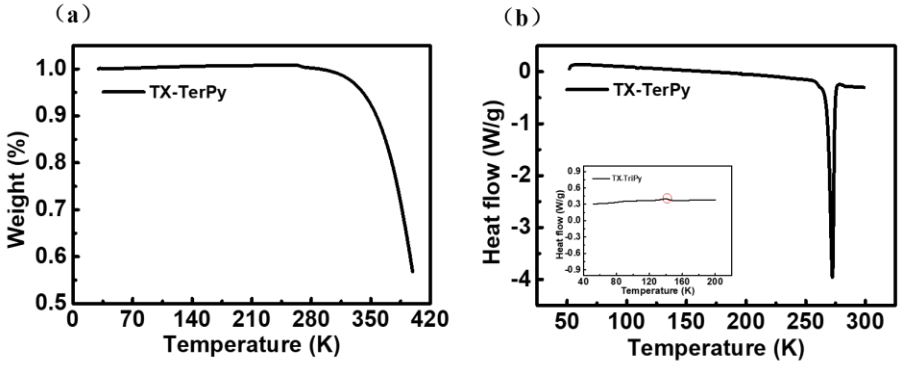

Figure S3. (a) TGA and (b) DSC curves of TX-TerPy.

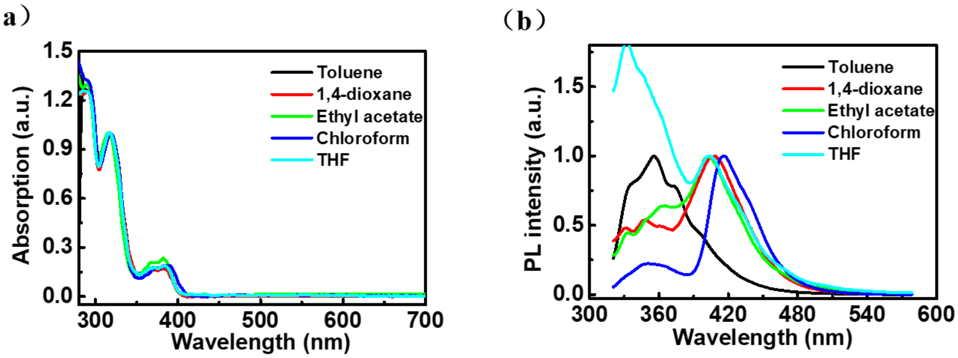

Figure S4. (a) Absorption spectra of TX-TerPy in different solvent; (b) PL spectra of TX-TerPy in different solvent. Concentration of TX-TerPy in all cases is 50 μM. The excitation emission is 300nm.

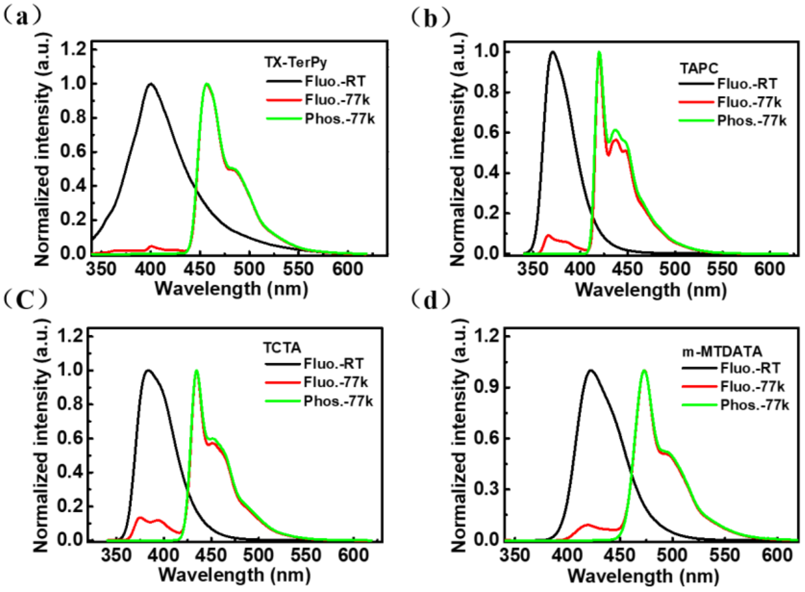

Figure S5. Fluorescence spectra and phosphorescence spectra in 2-MeTHF at room temperature and 77K (a) TX-TerPy; (b)TAPC; (c) TCTA; (d) m-MTDATA;

Table1. Summary of the excited state level of TX-TerPy, TAPC, TCTA and m-MTDATA in 2-MeTHF.

In solution	E_{S1}(RT) [a)] [eV]	E_{S1} (77k) [a)] [eV]	E_{T1} (77k) [a)] [eV]	ΔE_{ST} [b)] [eV]
TX-TerPy	3.09	3.09	2.71	0.38
TAPC	3.35	3.39	2.96	0.43
TCTA	3.23	3.15	2.85	0.30
m-MTDATA	2.94	2.96	2.62	0.34

[a)] Singlet (E_S) and triplet (E_T) excited energies estimated from the maximum wavelengths of fluorescence and phosphorescence spectra at room temperature and 77 K in 2-MeTHF,respectively; [b)] ΔE_{ST}=ΔE_S-ΔE_T.

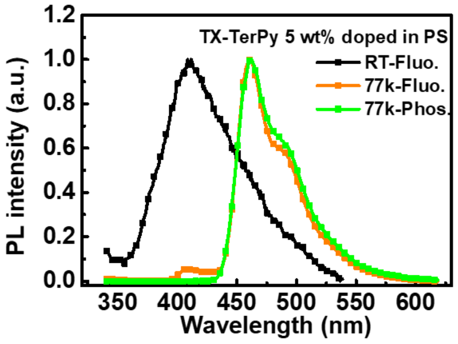

Figure S6. Fluorescence spectra and phosphorescence spectra in PS at room temperature and 77K of TX-TerPy.

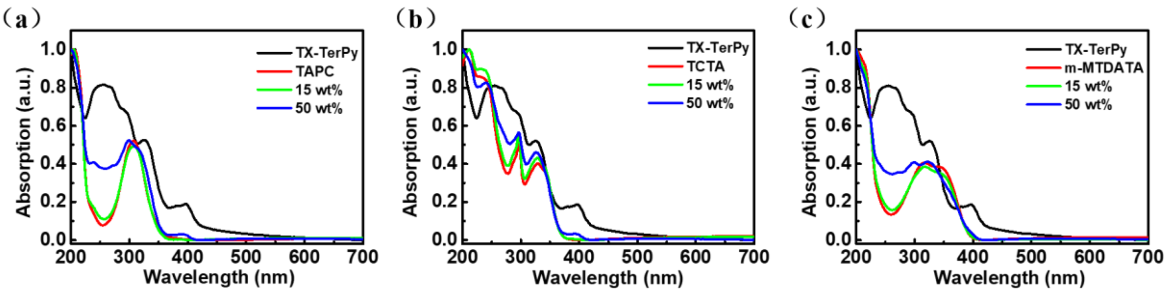

Figure S7. UV-Vis absorption spectra of deposited films. (a) UV-Vis absorption spectra of TX-TerPy, TAPC, TAPC:TX-TerPy (7:1) and TAPC:TX-TerPy (1:1); b) TX-TerPy, TCTA, TCTA:TX-TerPy (7:1) and TCTA:TX-TerPy (1:1); (c) TX-TerPy, m-MTDATA, m-MTDATA:TX-TerPy (7:1) and m-MTDATA:TX-TerPy (1:1).

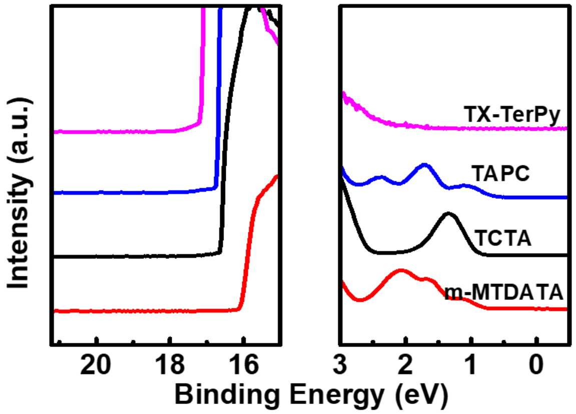

Figure S8. Ultraviolet photoelectron spectroscopy (UPS) of TX-TerPy, TAPC, TCTA and m-MTDATA pure film.

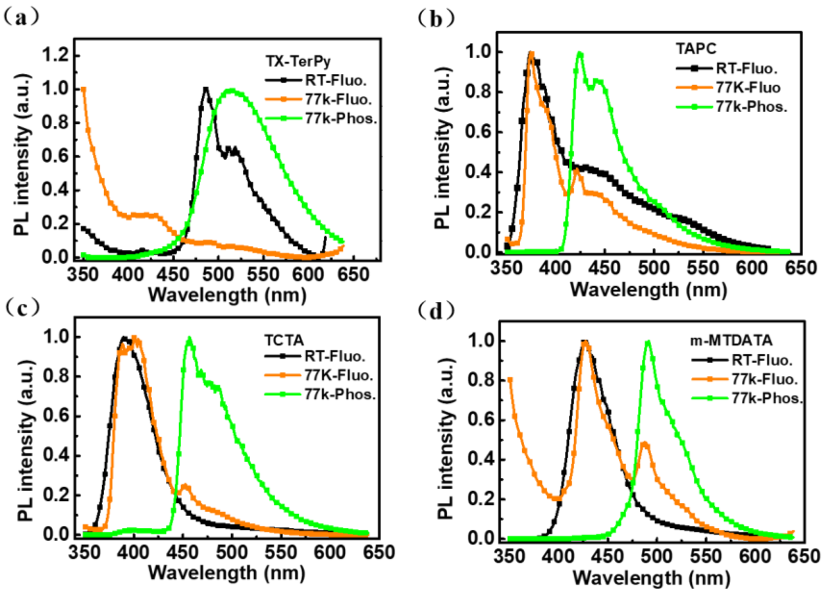

Figure S9. Fluorescence spectra and phosphorescence spectra in pure film at room temperature and 77K (a) TX-TerPy; (b)TAPC; (c) TCTA; (d) m-MTDATA.

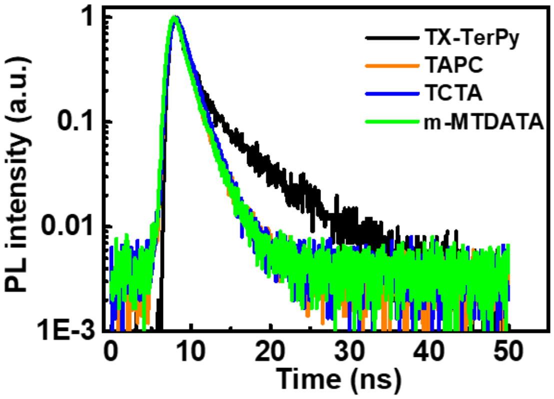

Figure S10. Transient PL decay of TX-TerPy, TAPC, TCTA, and m-MTDATA pure film in the time range of 50 ns.

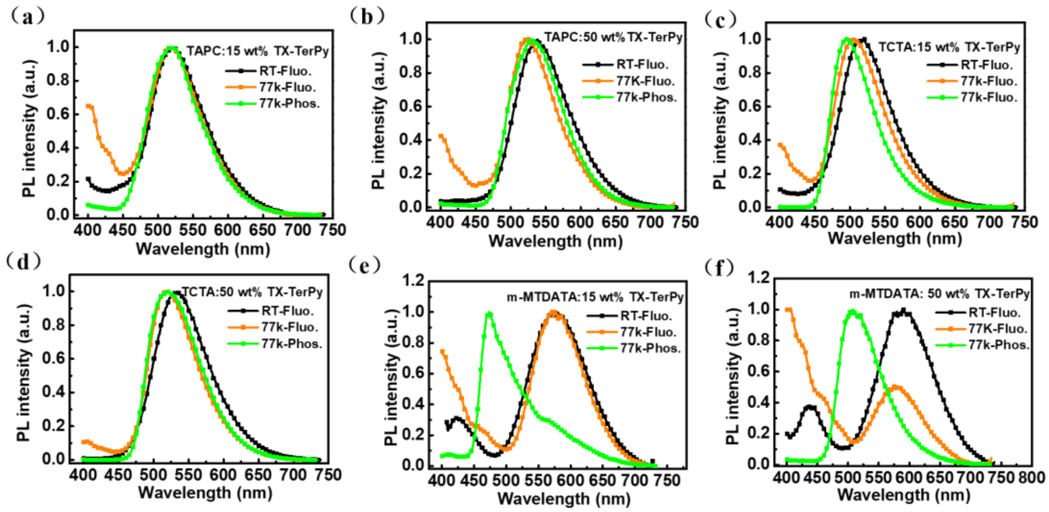

Figure S11. Fluorescence spectra of the doped film at room temperature and fluorescence spectra and phosphorescence spectra of the doped film at 77K; (a) TAPC:15 wt% TX-TerPy; (b) TAPC:50 wt% TX-TerPy; (c) TCTA:15 wt% TX-TerPy; (d) TCTA:50 wt% TX-TerPy; (e) m-MTDATA:15 wt% TX-TerPy; (f) m-MTDATA:50 wt% TX-TerPy.

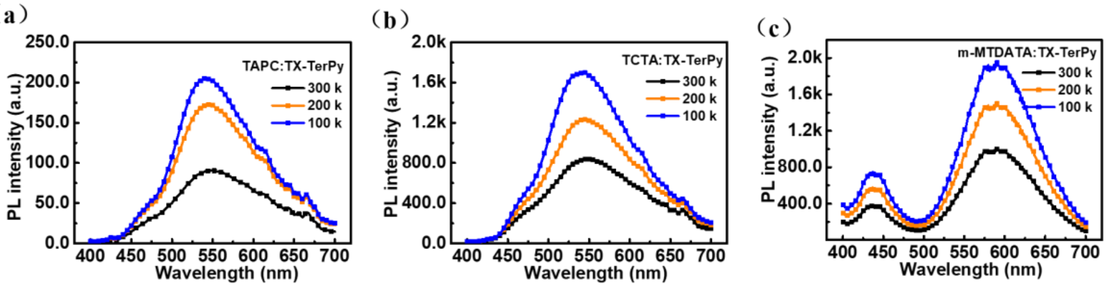

Figure S12. (a) PL spectra of TAPC:50 wt% TX-TerPy film at different temperature; (b) PL spectra of TCTA:50 wt% TX-TerPy film at different temperature; (c) PL spectra of m-MTDATA:50 wt% TX-TerPy film at different temperature.

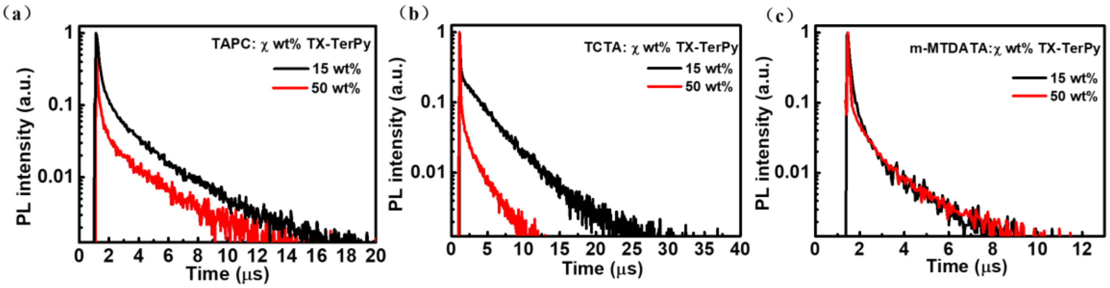

Figure S13. (a) Transient PL decay of TAPC:15% TX-TerPy and TAPC:50% TX-TerPy; (b) Transient PL decay of TCTA:15% TX-TerPy and TCTA:50% TX-TerPy; (c) Transient PL decay of m-MTDATA:15% TX-TerPy and m-MTDATA:50% TX-TerPy at room temperature in N_2 flow.

Table S2. Data extracted from the fluorescence spectra and phosphorescence spectra of the doped film and the corresponding individual molecule at 77K.

Acceptor	Donor	Conc.[a)] [wt %]	S_1[b)] [eV]	T_1[b)] [eV]
	Pure film	100	2.89	2.41
	In PS	5	3.03	2.69
	TAPC	0	3.31	2.93
		15	2.41	2.39
		50	2.36	2.34
TX-TerPy	TCTA	0	3.09	2.72
		15	2.50	2.46
		50	2.39	2.39
	m-MTDATA	0	2.90	2.52
		15	2.62	2.15
		50	2.45	2.15

a) Acceptor concentration. b) Singlet (E_S) and triplet (E_T) excited energies estimated from the maximum wavelengths of fluorescence and phosphorescence spectra at 77 K in the blend or pure film, respectively.
